# Molecular mechanics of *Staphylococcus aureus* adhesin, CNA, and the inhibition of bacterial adhesion by stretching collagen

**DOI:** 10.1371/journal.pone.0179601

**Published:** 2017-06-30

**Authors:** Ali Madani, Kiavash Garakani, Mohammad R. K. Mofrad

**Affiliations:** 1 Molecular Cell Biomechanics Laboratory, Departments of Bioengineering and Mechanical Engineering, University of California, Berkeley, California, United States of America; 2 Molecular Biophysics and Integrated Bioimaging Division, Lawrence Berkeley National Lab, Berkeley, California, United States of America; University of Florida, UNITED STATES

## Abstract

Bacterial adhesion to collagen, the most abundant protein in humans, is a critical step in the initiation and persistence of numerous bacterial infections. In this study, we explore the collagen binding mechanism of the multi-modular cell wall anchored collagen adhesin (CNA) in *Staphylococcus aureus* and examine how applied mechanical forces can modulate adhesion ability. The common structural-functional elements and domain organization of CNA are present across over 50 genera of bacteria. Through the use of molecular dynamics models and normal mode analysis, we shed light on the CNA’s structural and conformational dynamics and its interactions with collagen that lead to collagen binding. Our results suggest that the linker region, CNA_165-173_, acts as a hinge exhibiting bending, extensional, and torsional modes of structural flexibility and its residues are key in the interaction of the CNA-collagen complex. Steered molecular dynamics simulations were conducted with umbrella sampling. During the course of these simulations, the ‘locking’ latch from the CNA N2 domain was dissociated from its groove in the CNA N1 domain, implying the importance of the latch for effective ligand binding. Finally, we observed that the binding efficiency of the CNA N1-N2 domains to collagen decreases greatly with increasing tensile force application to the collagen peptides. Thus, CNA and similar adhesins might preferentially bind to sites in which collagen fibers are cleaved, such as in wounded, injured, or inflamed tissues, or in which the collagenous tissue is less mature. As alternative techniques for control of bacterial infection are in-demand due to the rise of bacterial antibiotic resistance, results from our computational studies with respect to the mechanoregulation of the collagen binding site may inspire new therapeutics and engineering solutions by mechanically preventing colonization and/or further pathogenesis.

## Introduction

The molecular pathogenesis of bacterial infections involves several different processes and factors. Cell wall-anchored proteins play critical roles in the pathogenesis of infections caused by numerous bacteria. To initiate host invasion, pathogens bind to extracellular matrix (ECM) proteins, which display a variety of specific adhesion sites for bacteria and eukaryotic cells. Many bacteria have evolved cell-surface proteins that expose recognition sequences for a majority of host ECM proteins, including collagen, fibronectin (Fn), fibrinogen (Fg), and other proteins [1]. Recently, mechanical factors have been implicated in the regulation of bacterial adhesion to the ECM. For fibronectin-binding bacterial proteins, it has been shown that stretching fibronectin fibers can disrupt bacterial adhesion. [2]

Collagen is a class of proteins that provides the structural support for tissues, serves as a scaffold for the assembly of the ECM, and can also directly affect cell behavior through specific cellular receptors. Over twenty different collagen types have been identified, not including a number of proteins that contain collagenous subdomains. Collagen has a characteristic triple helix structure with polypeptides composed of repeating Gly-X-Y sequences that form hetero or homo-trimeric L-proline helices. It is not surprising that many bacteria have evolved to produce collagen adhesins to interact with this group of proteins. As demonstrated by Staphylococcus aureus collagen adhesin (CNA) [3–6] and Yersinia *enterocolitica* adhesin A (YadA) [7] in various animal models, these interactions can be influential in the establishment and progression of bacterial infections. For example, mice infected with *S*. *aureus* strains expressing CNA initially had similar numbers of *S*. *aureus* as mice infected with an isogenic *S*. *aureus* strain that expressed a mutated inactive form of CNA. However, the former group of mice showed significantly more *S*. *aureus* microbes than the latter group as the infection progressed—within less than a 24 hour post-inoculation period [8]. Therefore, the collagen adhesins allow the bacteria to adhere strongly enough to tissue structures containing their corresponding ligand in order to resist clearance by the host defense system. The collagen adhesins affect infections in various tissue structures in the body. The cardiovascular system has been shown to be affected by bacterial collagen adhesins—particularly *Enterococcus faecalis* adhesin, ACE, and *Enterococcus faecium* adhesin, ACM, contributing to disease progression in experimental endocarditis. [9,10] *Streptococcus mutans-*expressed protein CNM enables adhesion to heart endothelial cells [11] and is associated with hemorrhagic stroke [12].

Among the collagen adhesins of bacteria, CNA of *S*. *aureus* has been characterized more thoroughly. CNA is a cell wall-anchored protein that belongs to the MSCRAMM (microbial surface component recognizing adhesive matrix molecules) family of adhesins. CNA participates in the infectious process of pathogenic *S*. *aureus* and is shown to be a virulence factor in many different animal models of staphylococcal infections including arthritis, keratitis, endocarditis, mastitis, and osteomyelitis [3–5,13,14]—demonstrating that the ability to interact with collagen provides a general advantage to the bacteria in pathogenesis. Furthermore, the recombinant CNA can even be designed as an effective vaccine component and antibodies raised against CNA are protective in a mouse model of *S*. *aureus* induced septic death [15].

Similar to the domain organization typical of MSCRAMMs, CNA has a signal peptide sequence at the N terminus, a non-repetitive A region, followed by a varying amount of B repeats, and a cell wall-anchoring region including an LPXTG-motif, a transmembrane segment, and a short cytoplasmic tail rich in positively charged residues. Within the CNA A region consists three subdomains: N1, N2, and N3. Of particular interest, the N2 subdomain, a 168-amino-acid-long segment, has been characterized as the minimum collagen binding region [3]. The crystal structure of the N2 subdomain shows its IgG-like folds, composed of two antiparallel β-sheets and two short α-helices. By molecular protein docking software, a shallow groove on one of the β-sheets of N2 was identified as a putative binding interface [16]. Studies later showed that the N1-N2 domains bind to collagen with a considerably higher affinity than the N2 domain alone or the intact A region [17]. The crystal structures of the N1-N2 segment of CNA, both as an apo-protein and in complex with a synthetic collagen triple helical peptide, have been analyzed and led to the postulation of a Collagen Hug mechanism for binding [17]. According to this mode, the N1-N2 subdomains of CNA adopt an open conformation where a binding trench on the N2 subdomain is accessible, allowing the collagen triple helical ligand to dock. As a result, the CNA wraps around collagen and creates a tunnel-like structure that stabilizes the collagen ligand in between the two N1-N2 subdomains. In a final step, the C-terminal extension of the N2 subdomain acts as a latch by inserting into a trench present on the N1 subdomain by β-strand complementation—effectively locking the complex in place [17].

**Fig 1:**
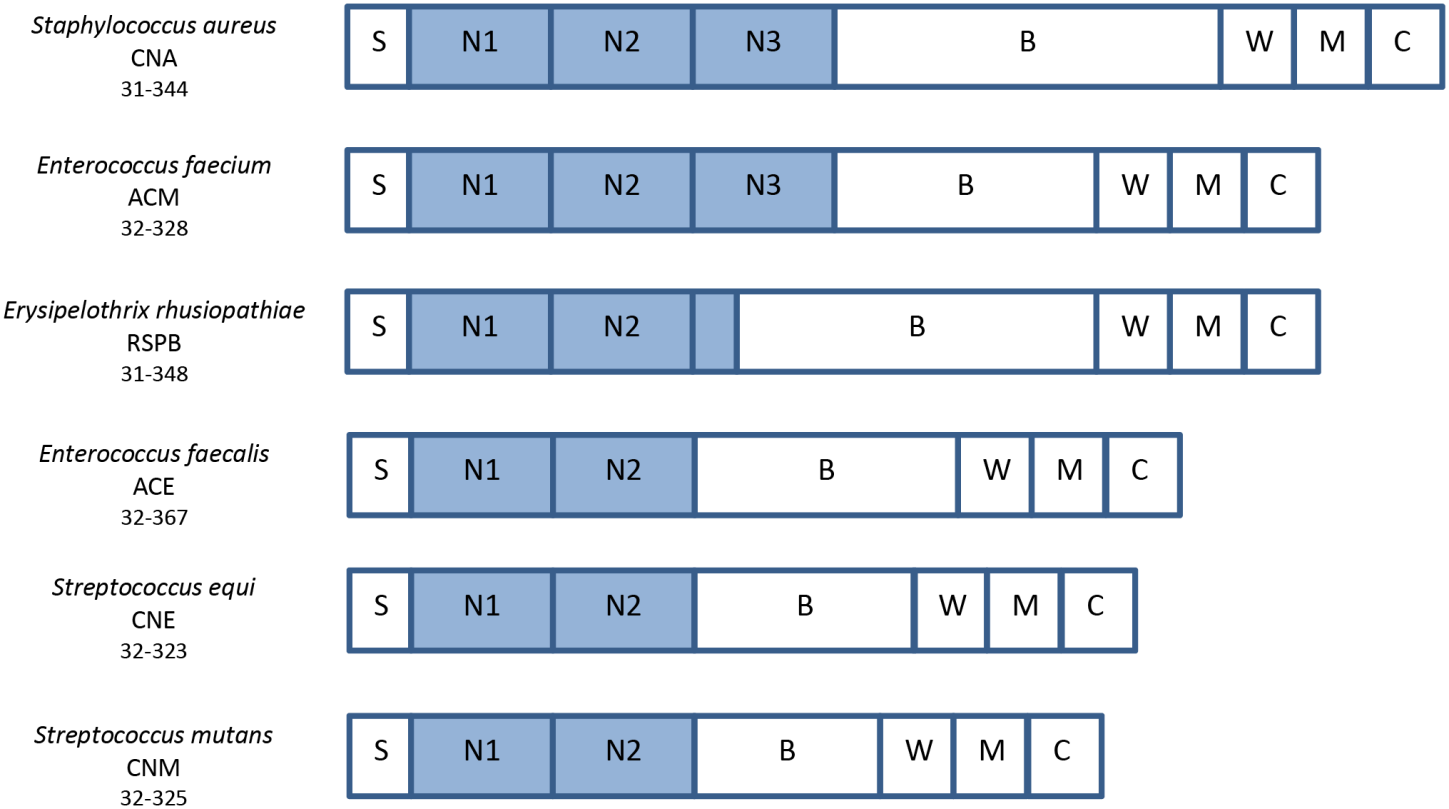
Similar domain organization of crystallized collagen-binding MSCRAMM adhesins. The collagen-binding adhesin of *Staphylococcus aureus*, CNA, has similar domain structure as many other bacterial adhesins. Therefore, CNA serves as a model protein that will share functional binding characteristics as other bacteria. The following are abbreviated: S—signal peptide; N1, N2, N3—collagen-binding region; B—repeats region; W—cell wall-anchoring region; M—trans-membrane region; and C—cytoplasmic tail. As evident, CNA is a characteristic adhesin in which similar proteins can be found across many species of bacteria.

The domain architecture present in members of the CNA-like family of collagen-binding MSCRAMMs is shown in Fig 1. Through experimental and crystallographic verification these proteins have been shown to exhibit similar structure and function, and are found in many Gram-positive bacterial species, i.e. ACE in *Enterococcus faecalis* [18], ACM in *Enterococcus faecium* [19], CNE in *Streptococcus equi* [20], CNM in *Streptococcus mutans* [21], and RSPB in *Erysipelothrix rhusiopathiae* [22]. These collagen-binding MSCRAMMs are virulence factors in human and animal infectious diseases and mediate bacterial attachment to collagen-rich tissues.

Many open questions exist regarding the pathogenesis of collagenous tissue. Mainly, what is the role of mechanical factors on regulation of bacterial adhesion to collagen? At molecular-scale granularity, what insights can we draw from the binding mechanism of CNA-Collagen adhesion and how can mechanical forces potentially modulate these binding interactions?

The objective of present study is to use bioinformatics and computational biophysics approaches to shed light on the molecular details of collagen binding mechanism of CNA in S. Aureus. We study CNA’s structural and conformational dynamics and its interactions and binding with collagen. Using a combination of molecular dynamics (MD) simulations with umbrella sampling and normal-mode analysis (NMA), we explore the molecular details of the Collagen Hug Model for binding mechanism [17] and examine how adhesion is modulated by stretching collagen proteins. Our results suggest that bacterial peptides bind significantly less to stretched collagen as compared to relaxed collagen, thus demonstrating the mechanoregulation of a bacterial binding site on collagen.

## Results

A basic bioinformatics analysis was conducted of the two globular subdomains separated by a linker to observe how ubiquitous the structural domains and associated binding mechanism are in nature. The residue sequence of the N1-N2 CNA domains were extracted from the crystal structure (2F6A) provided by the RCSB PDB and verified by the Uniprot database. A sequence similarity search was then performed using the FASTA algorithm to find homologous proteins in bacteria, eukaryota, viruses, and archaea. For our considerations, we examined proteins with greater than 20% identity and with expectation values below 10 as otherwise the homology would not be considered biologically significant. Other considerations such as the length of the segment similar between the two sequences and the number of insertion/deletions were taken into account.

Using the UniProtKB [23] taxonomic databases, we found numerous homologous proteins in nature. For bacteria, there were proteins spanning 51 different genera, 110 different species, and 864 different strains that fit our set criteria. For eukaryota, archaea, and viruses, we found 11, 13, and 11 similar proteins, respectively. Compared to bacteria, the quality of the matches were significantly lower, however out of the three domains, archaea had some proteins with E-values less than 0.1.

### CNA-collagen binding is a combination of multiple, specific interaction effects

**Fig 2:**
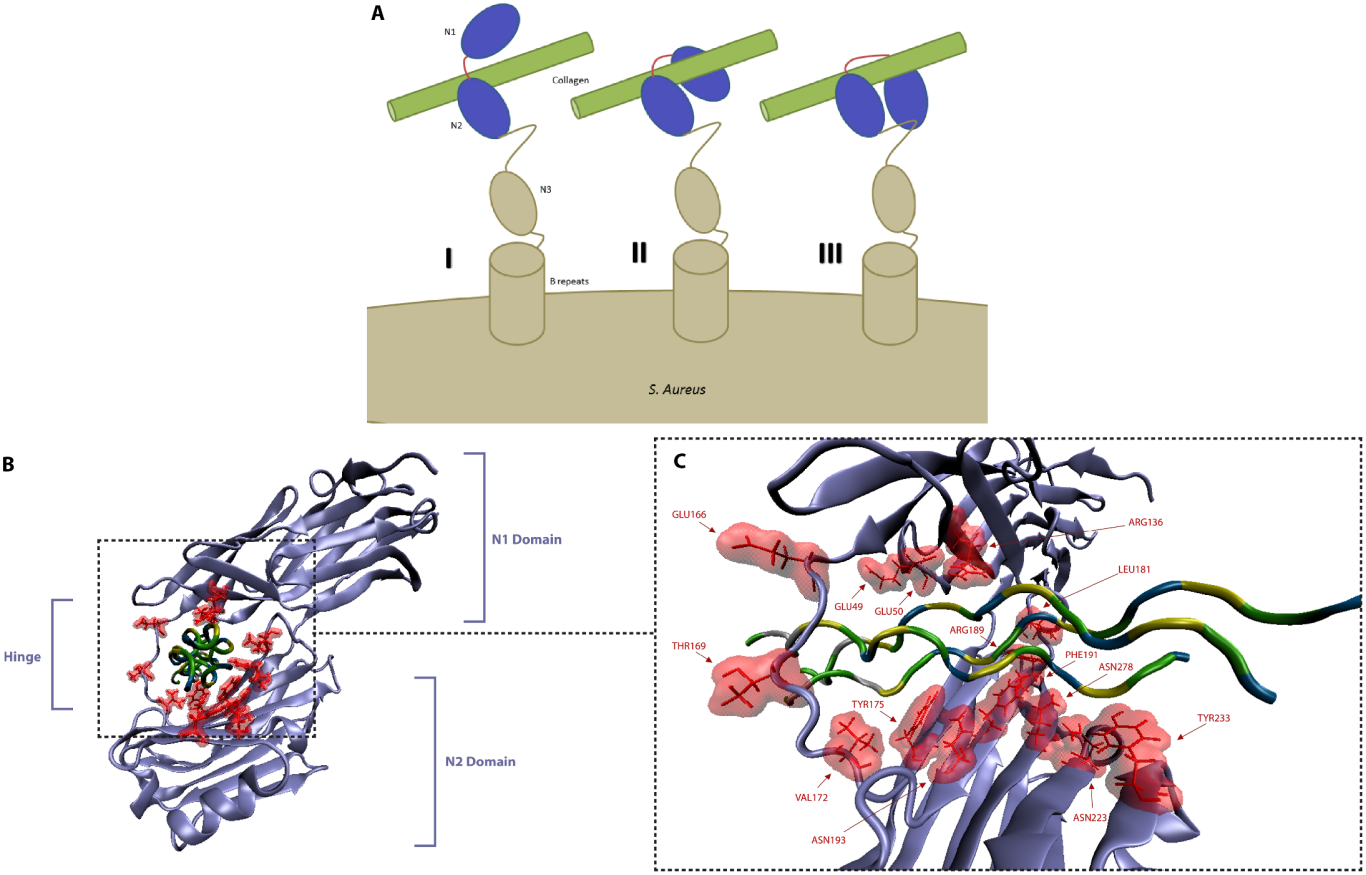
Binding mechanism of bacterial adhesin, CNA, with extracellular matrix protein, collagen. (A) Hypothetical “Collagen Hug” schematic [17] demonstrating the (i) open state with initial collagen association with the N2 domain (ii) wrapping of collagen by the linker and N1 domain (iii) final closed state with the N1 domain interacting with the N2 domain and also the latch region secured in a groove in the N1 domain. (B) The CNA-collagen complex. The N1 and N2 globular domains along with their linker/hinge are shown wrapped around collagen. (C) Particular residues in CNA involved in interaction with collagen are highlighted and labeled. The collagen peptides are segmented by residue type with basic, non-polar, polar, and hydroxyproline residues colored as silver, yellow, green, and blue respectively.

The Collagen Hug model [18] describes a process in which the N1-N2 domains of CNA adopt an open conformation, enabling collagen binding to a present trench in CNA, followed by a locking latch strand as shown schematically in Fig 2A. There is a multitude of specific interaction effects present in the CNA-Collagen complex as investigated below.

Each CNA_31-344_ molecule interacts with three and a half GPO repeats at each end of the collagen peptide. The non-covalent interactions attributing to the overall conformational structure include hydrophobic, electrostatic, and van der Waals interactions. Within these, we observed for each peptide chain in collagen there is one site for salt bridges. For the Arg15, Arg15, and Arg17 residues in the three collagen peptides, we observed three salt bridges formed with residues Glu49, Glu166, and Glu166 on CNA respectively. In addition, there were many hydrogen bonds formed with varying occupancy levels. The CNA residues Asp 50, Tyr175, Arg189, Gly168, Thr169, Glu166, Glu166, Asn196, Asp179 formed hydrogen bonds with Hyp12, Hyp12, Hyp9, Hyp12, Hyp12, Arg15, Arg17, Hyp9, Hyp12 respectively.

Most of the CNA-collagen interactions involve residues in a trench on the N2 domain of CNA_31-344_, which is holding the leading and trailing chains of the collagen peptide. Although the direction of the bound ligand is about 30° rotated from an alternately proposed trench-docking model [16], most of the trench area is covered by the collagen peptides. Our results confirmed that all of the trench residues previously implicated in collagen binding [16] are in fact contacting the collagen peptide. In addition to the interactions between residues in the N2 domain and the leading and trailing chains of collagen as shown in Fig 2C, there are two additional hydrophobic residues, Leu181 and Val172, which together are important for ligand-locking. Consequently, Phe191 and Tyr175, hydrophobic residues present in the N2 domain trench region, are stacked against the hydrophobic Pro8 and Pro11 of the leading chain. Also stacked against each other are Tyr233 and Pro5 of the trailing chain, and the polar residues Asn193, Asn223 and Asn278 in the trench region are hydrogen bonded to trailing chain Hyp9 and Hyp12 residues.

Compared to the N2 subdomain, the N1 subdomain of CNA_31-344_ displays limited interactions with collagen. Of special note, the CNA_164-173_ linker seems to be playing an important role in holding the ligand in place by interacting with proline residues in the leading and trailing chains. The Asp50 and Arg136 residues of the N1 domain interact with Hyp6 of the middle chain and Hyp9 of the trailing chain. Notably, the Pro11 from the leading chain is situated between the Val172 residue from the inter-domain linker and Tyr175 from the N2 domain. This has been suggested to affect conformational changes leading to sequestration of the triple helical collagen [17].

### CNA latch dissociation requires energy by active processes

**Fig 3:**
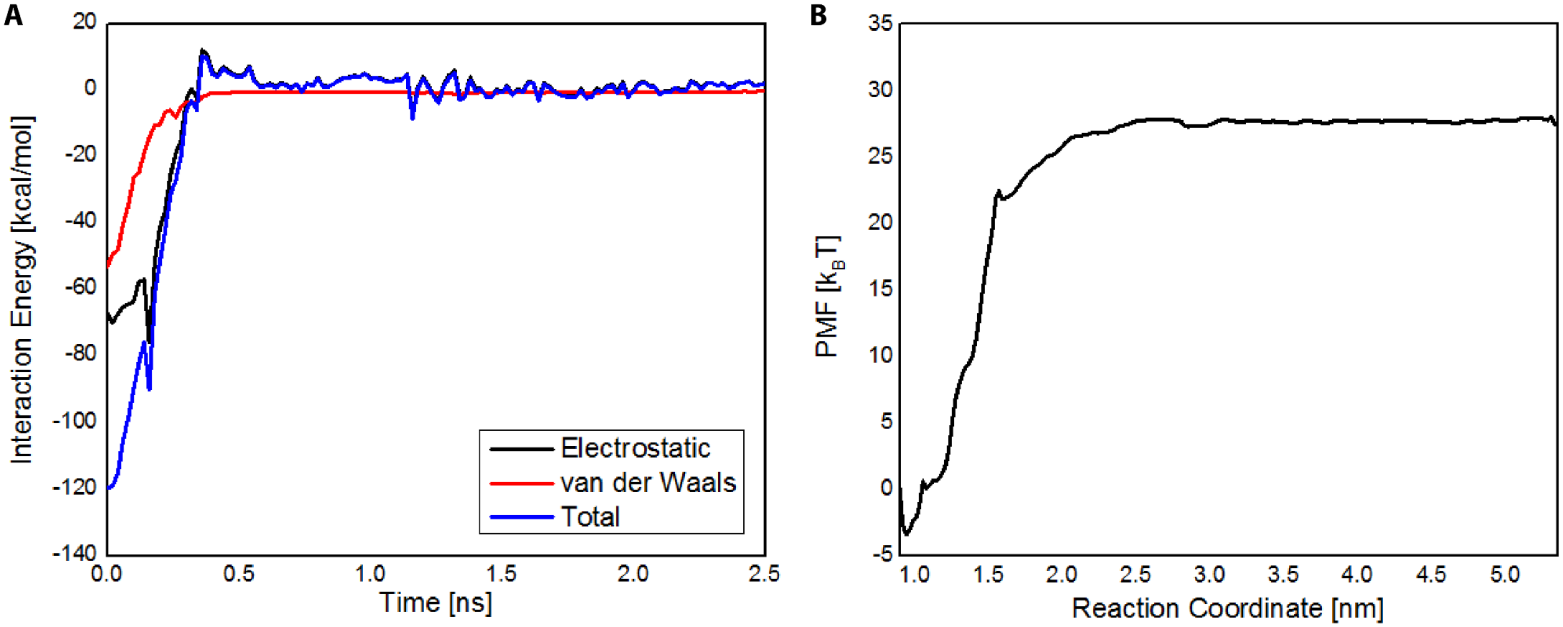
CNA N2 latch dissociation from ridge in N1 domain requires larger energy than provided by thermal energy. (A) Electrostatic, van der Waals, and total interaction energy is shown for the Latch—N1 domain interaction. (B) Potential mean force (PMF) profile of latch dissociation from N1 domain. N2 latch dissociation from N1 domain was endothermic, requiring nearly 28 k_B_T of free energy—indicating that latch dissociation may require active processes and not solely through surrounding thermal energy.

To analyze the interaction between the N2 latch and N1 domain of CNA, we performed steered molecular dynamics simulations coupled with umbrella sampling of the latch and N1 domain. Shown in Fig 3A is the calculated interaction energy between the latch and N1 over the course of the latch constant force pulling. As expected, the interaction energy between the latch and N1 decreased to zero over the course of the simulations. As seen in Fig 3A, the interaction energy between the latch and N2 in the closed CNA was approximately 120 kcal/mol, and consisted of both van der Waals and electrostatic interactions.

To determine whether the latch may undergo dissociation from N1 solely from energy provided by thermal fluctuations, we performed umbrella sampling simulations to calculate the potential mean force (PMF) profile of latch dissociation from the N1 domain (Fig 3B). The reaction coordinate was defined as the distance between the center of masses of the N1 domain and the N2 latch, where lower values corresponded to latch-associated N1, and higher values corresponded to dissociated N1. Besides the N2 latch, the N2 domain was excluded in umbrella sampling simulations. As seen in Fig 3B, N2 latch dissociation from N1 was endothermic, and required ~28 k_B_T of free energy, indicating that thermal energy is not sufficient to significantly dissociate the latch from the N2 domain of CNA and supporting the notion that N2 latch dissociation from the N1 domain may require active processes.

### CNA N1-N2 domains exhibit natural modes that confirm collagen ‘hugging’

**Fig 4:**
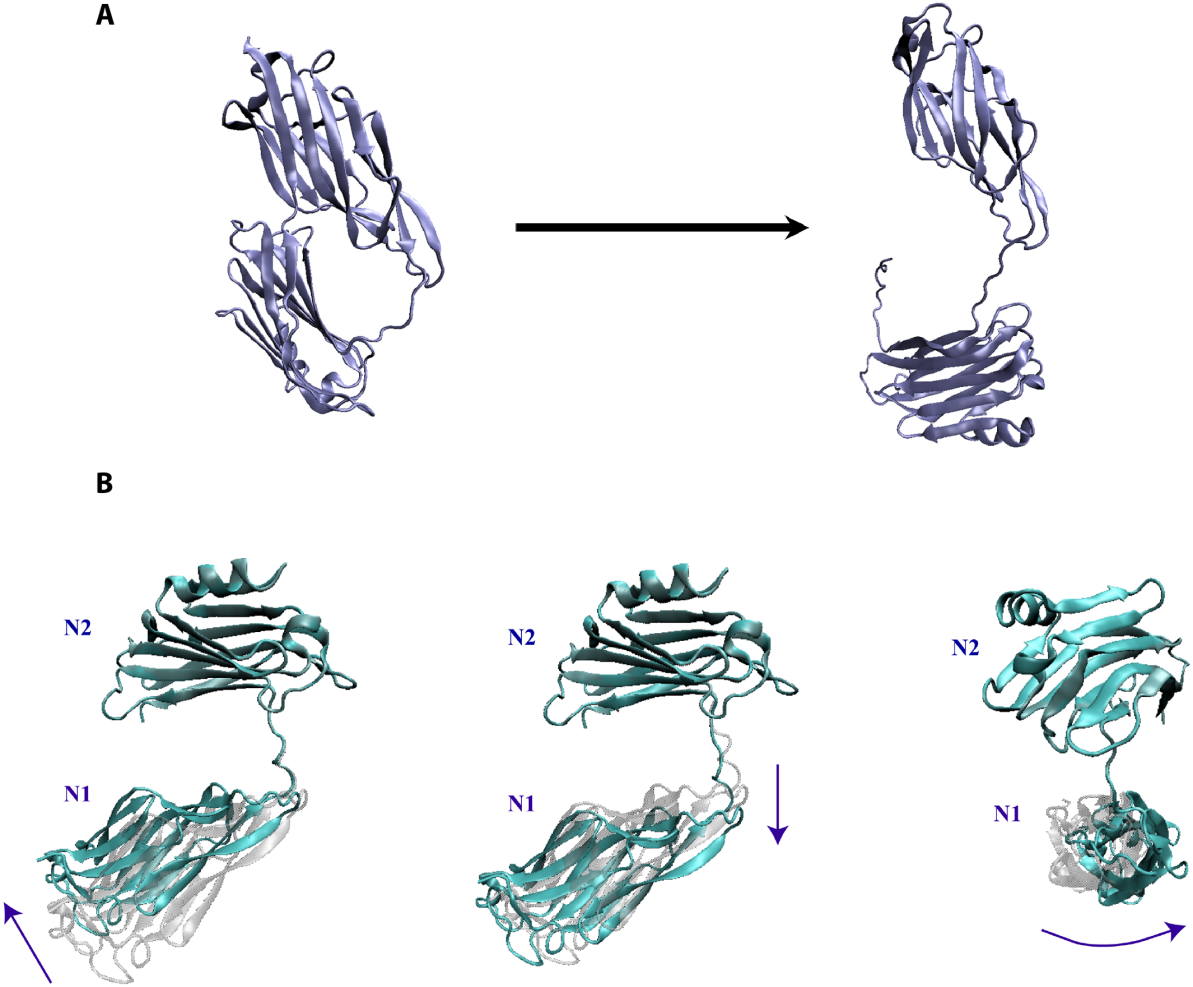
Inducing an *in silico* open-state CNA and demonstrating the natural frequencies of the CNA N1-N2 domains. (A) The open conformation of CNA (right) was obtained by performing Steered Molecular Dynamics on the closed CNA conformation (left). (B) After equilibrating the open-state CNA, normal mode analysis on the CNA domains revealed modes with bending, twisting, and extending motions. Shown are the three lowest vibrational frequency modes that are the distinct modes of movement indicated by the arrows. The linker region between the N1 and N2 domains serves as a highly flexible hinge.

The Collagen Hug model (see Fig 2A) proposes the existence of an open-state CNA conformation necessary for collagen binding. To quantitatively analyze the detailed interactions involved in the Collagen Hug model and to perform the subsequent normal mode analysis, we generated an *in silico* open structure of CNA using steered molecular dynamics. Fig 4A shows the initial and final states after applying forces to disassociate the latch region from the N2 domain and separate the interacting N1 and N2 domains of CNA with the necessary equilibration post-SMD.

Structural properties of the N1-N2 domains of CNA can be inferred from its natural vibrations. Therefore, to reveal the naturally rigid and flexible regions of CNA, we carried out normal mode analysis. Results from NMA convey properties inherent in the structure of CNA regardless of what intermolecular interactions are present [24]. The purpose of our NMA was to determine the contributions of the CNA structure to its mechanical behavior. This would shed light on possible models for conformational changes that contribute toward collagen adhesion. NMA was carried out on both the closed state with no collagen present and the *in silico* open state induced by our steered molecular dynamics and equilibration simulations. The NMA results suggest CNA to have natural bending, extensional, and torsional flexibility that fits well with the Collagen Hug Model.

NMA from the open state CNA was more informative than the closed state CNA toward understanding the binding mechanism with collagen. The closed state exhibited limited structural flexibility as to not suggest any likely opportunities for adhesion to collagen while closed. The *in silico* open state CNA exhibited significant binding flexibility and some torsional flexibility can shed light into plausible mechanisms for collagen binding.

Due to the multi-modular structure of the protein, in which it exhibits IgG-like folded domains separated by linker peptides, the linker region in CNA from residues 165 to 173 is critically important to its natural conformational modes of movement. The NMA analysis suggests that the linker region serves as a highly flexible and dynamic hinge providing a variety of available movements. Excluding the rigid-body translational and rotational modes, we observed the three lowest and representative modes of movement in Fig 4B. The first mode shows a bending hinge movement that would most closely resemble the structural dynamics enabling the Collagen Hug Model as drawn in Fig 2A. The second mode exhibits the extension flexibility of the coiled linker region. In addition, it could indicate mutagenesis or addition/deletion events in the linker region could greatly affect the structural dynamics involved in collagen. The last mode exhibits the torsional movement that further expands the range of movement associated with the two domains. In all representations, the N2 domain is aligned to be held constant in visualization as the N1 domain is situated furthest from the cell wall. These NMA results were validated by long-term equilibration of the system by molecular dynamic simulations and observing mechanical modes of movement by CNA. The combination of NMA and MD simulations provide a convincing picture for molecular mechanics of the collagen-binding protein.

### Mechanoregulation of CNA binding to collagen under various tensile forces

**Fig 5:**
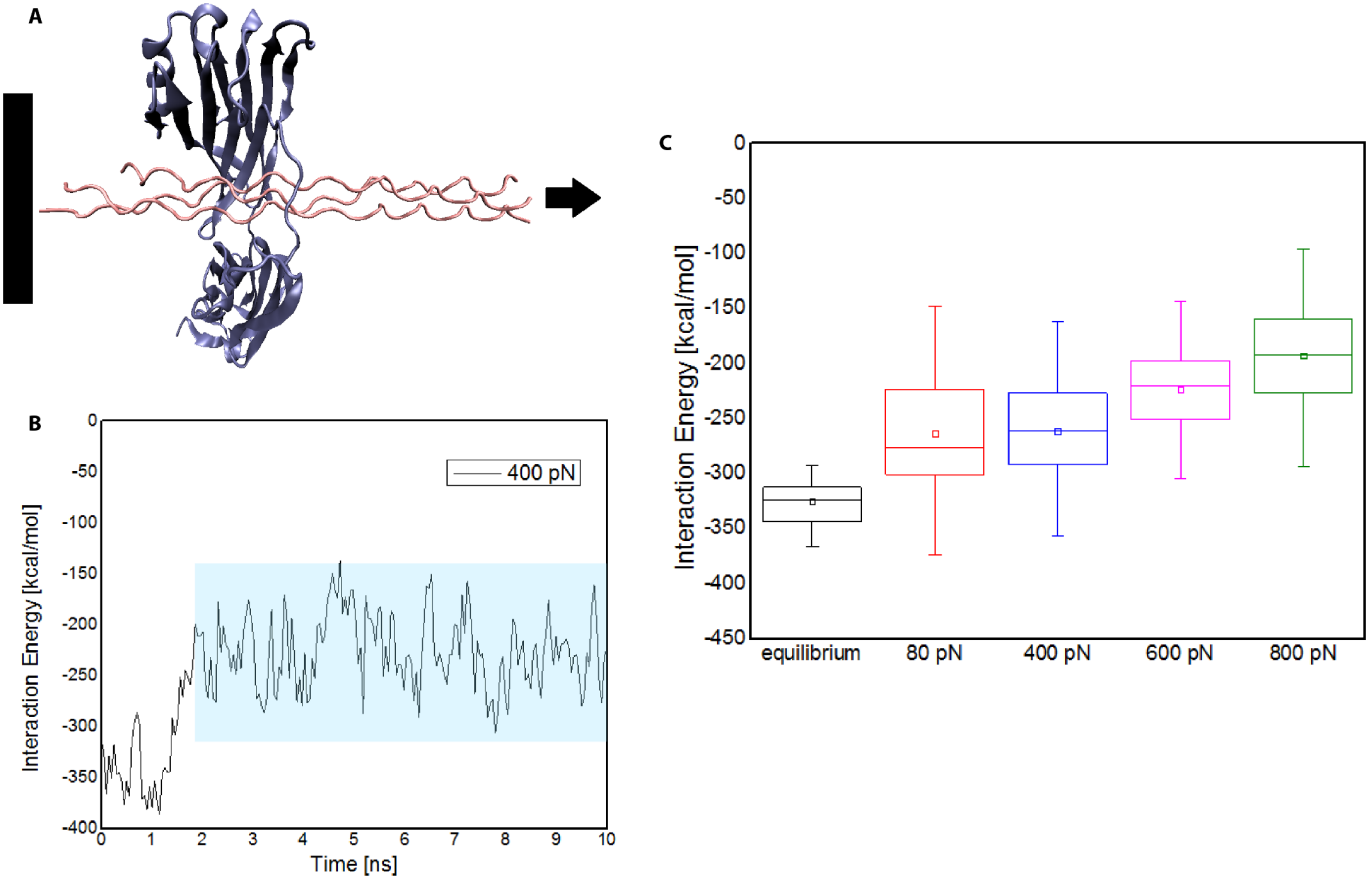
Mechanoregulation of bacterial binding to collagen via applied tensile load. (A) SMD simulation illustrating the tensile force applied to collagen while in complex with the N1-N2 domains of CNA. (B) Plot of an individual SMD simulation trial, in this case with 400 pN load, for interaction energy between the CNA N1-N2 domains and collagen. The data is sampled once the interaction energy stabilizes—region denoted by the shaded box. These windows in aggregate are displayed in part C. (C) Distribution of interaction energies for the CNA-collagen complex in equilibrium and under 80 pN, 400 pN, 600 pN, and 800 pN of tensile force applied to collagen. Results suggest that susceptibility of bacterial adhesion to collagen decreases under higher tensile forces in the body.

To explore the underpinning mechanism by which tensile force exerted on collagen may disrupt bacterial binding sites, we used steered molecular dynamics to simulate the stretching of collagen in complex with CNA of *Staphyloccocus Aureus*. As shown in Fig 5A, the collagen peptides were fixed at one end and a constant pulling force was applied at the other end. A range of stretching forces were applied on the collagen while its interactions with CNA were examined. Given the right-handed helical structure of collagen, the peptides uncoiled in a characteristic torsional manner. The rate and extent of uncoiling was directly related to the magnitude of force applied.

The interaction energy between the CNA N1-N2 domains and collagen was measured for each stretching force scenario. After the initial stretching and uncoiling of the collagen peptides, the interaction energy decreases in magnitude and oscillates near an equilibrium binding energy value as shown in Fig 5B. For statistical significance, we repeated the simulation three times for every force level. Also to ensure stabilization of the interaction energy for longer simulation times, we ran two systems for 40 ns—both of which confirmed a stable interaction energy plot. The distribution of total interaction energy between CNA and collagen at equilibrium and under varying levels tensile force applied to collagen were studied (see Fig 5C). We observe the total interaction energy between CNA and collagen is comparable at 80pN and 400pN force levels but reduces as the stretching force increases beyond 600pN. The structural visualizations in VMD also confirm that the non-covalent interactions decrease due to the force-sensitive rearrangements in collagen secondary structure. The interaction energies and visualizations imply that under higher tensile forces on collagen in the body, bacterial adhesion, and subsequent infection, may be less likely.

We also conducted SMD simulations for forces greater than 800 pN, namely 4000 pN and 8000 pN, to investigate the upper bound of forces. Similar to other ECM proteins, collagen is consistently subject to mechanical stress in the ECM; however, it would be best to include only physiologically relevant forces. In examining the results with VMD, we notice significant structural changes at 8000 pN as to suggest protein denaturation and thereby not relevant.

**Fig 6:**
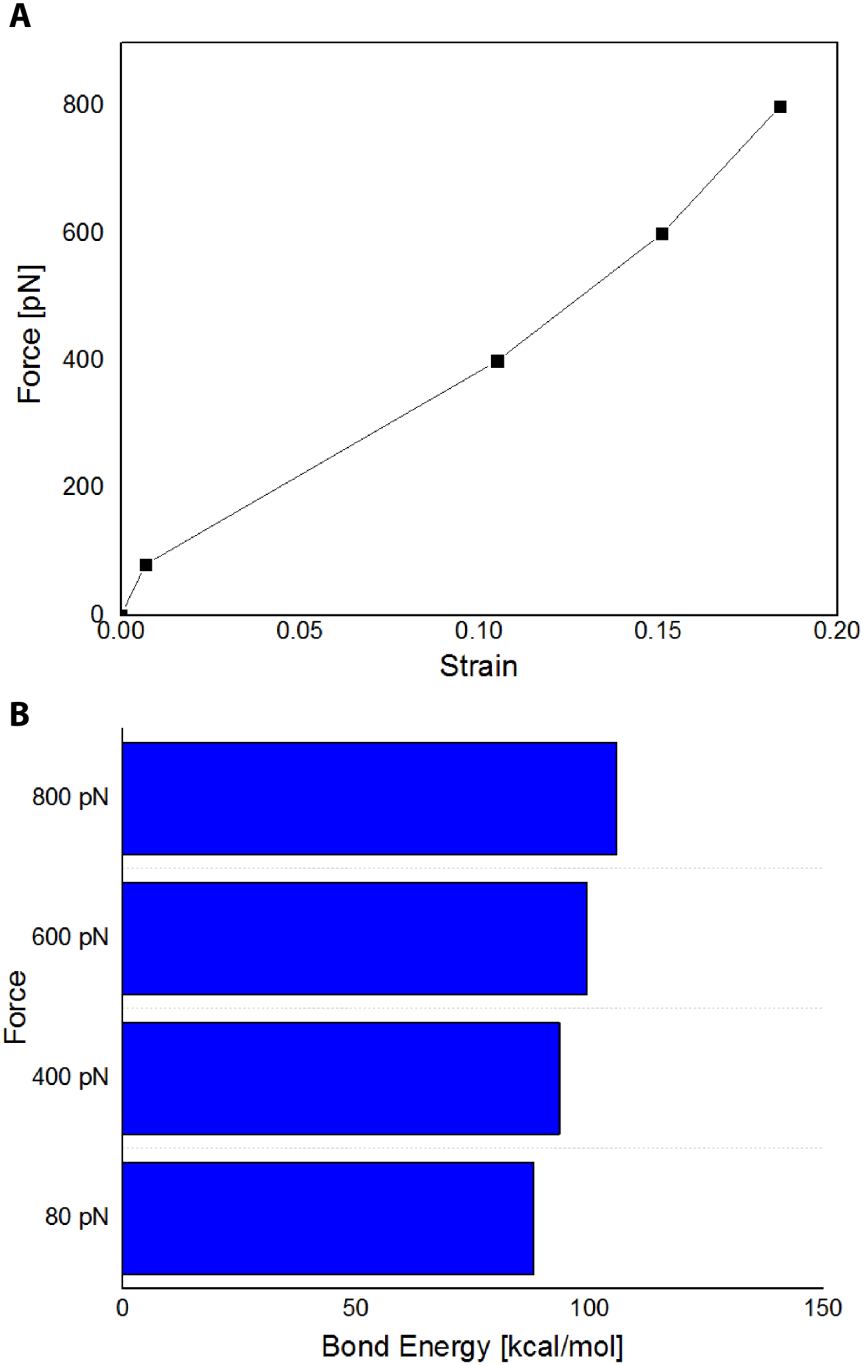
Verification of the biological relevance of applied tensile forces. (A) The average strain vs. load curve demonstrating the force dependence on length elongation of collagen. (B) Calculated average bond energies for the individual collagen peptides.

Next, we examined the load-strain behavior of the collagen model during the SMD simulations (see Fig 6A). The load-strain curve for forces 800 pN and less is fairly linear before parting its initial linear regime to non-linear stiffening governed by the elasticity of each covalent bond—thereby indicating physiological relevance of the force. Similarly, the bond energy calculations for individual collagen peptides (see Fig 6B) also show a linear relationship with applied force. In most biologically relevant cases, the forces applied will only lead to the disruption of non-bonded interactions that lead to higher-order changes in protein structure. From the bond energy calculations for the individual collagen peptides (see S1 Fig), it is shown that the 4000 pN and 8000 pN exert non-linear behavior strain on the collagen peptide itself—pulling the residues apart. As molecular dynamics does not allow for breaking of covalent bonds, collagen proteins, with similar mechanical properties to collagen I in the body, at forces of 4000 pN and 8000 pN will approach mechanical failure, and thus these forces are likely not biologically relevant.

**Fig 7:**
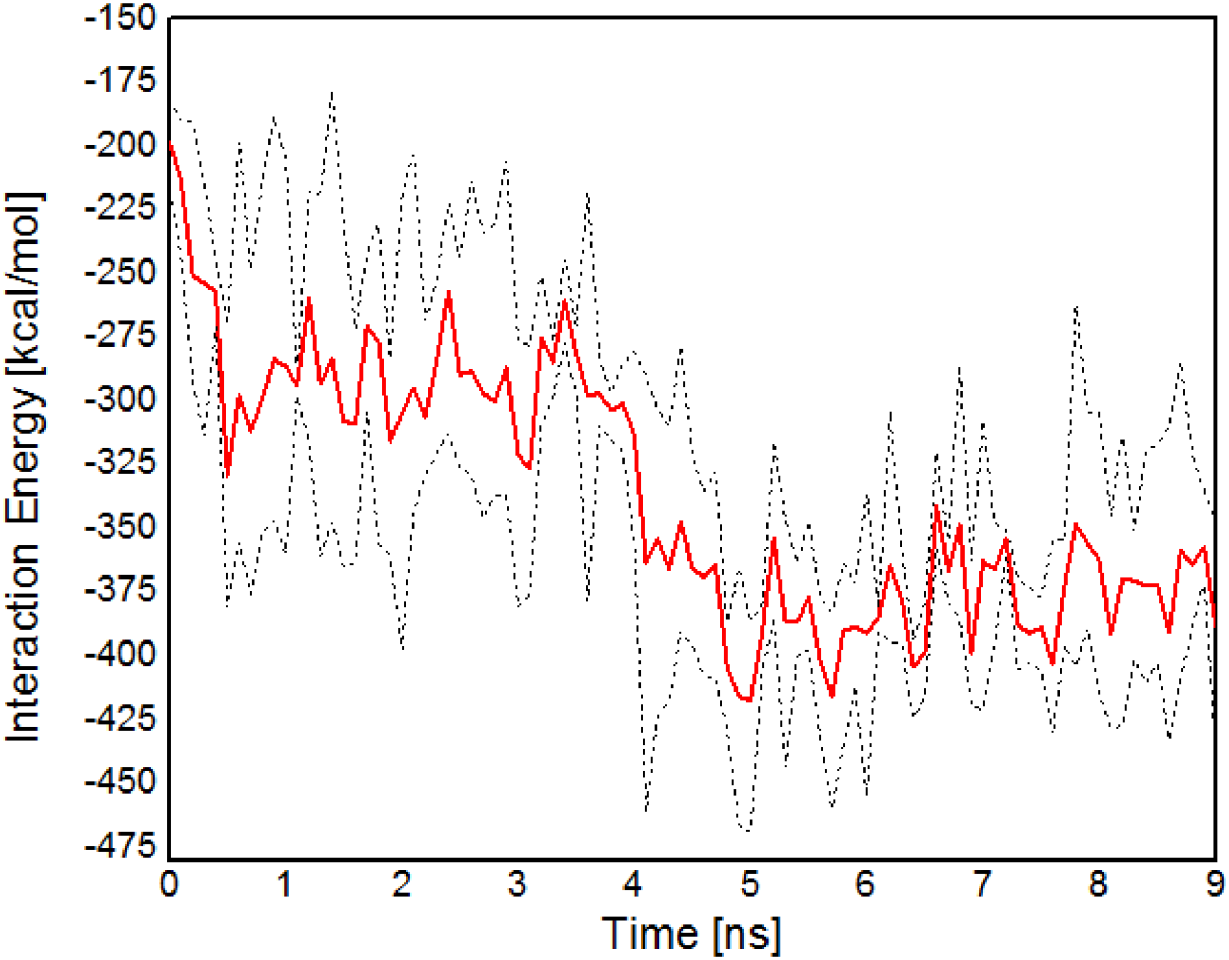
Reversibility of collagen binding post-release of 800 pN force on collagen. The red line plots the average interaction energy value for multiple trials and the dotted lines demonstrate maximum and minimum boundaries. The graph implies that the higher end of forces in the study is physiologically feasible.

Finally, the reversibility of CNA-collagen binding post-release of applied force on collagen was examined. For the 800 pN applied force simulations, we ran three separate trials examining what structural changes occur after releasing the load. As shown in Fig 7, the average interaction energy increased in magnitude with time. As the collagen peptides recoil, re-enabling binding sites within the N1-N2 CNA domains, and electrostatic interactions are increased, the total interaction between the CNA-collagen complex is strengthened. This indicates for the higher end of forces, 800 pN, the CNA domains reversibly bound to collagen for all three separate simulations once the load was released—denoting little denaturation of the original collagen protein due to higher tension.

## Discussion

Bacterial adhesion onto ECM proteins is one of the key steps in pathogenic invasion and infection. [25] Collagen, an ECM protein with a characteristic triple helix structure, is the most abundant protein in mammals, making up to 25% to 35% of the whole-body protein content. [26] It is present in variety of regions in the human body, particularly connective tissues, and many bacteria have evolved to produce collagen adhesins. One of these collagen adhesins, CNA in *Staphylococcus Aureus*, is a multi-modular protein spanning the ECM, cell wall, membrane, and cytoplasm of bacteria. The crystal structure of the N1-N2 segment of CNA, both as an apo-protein and in complex with a collagen triple helical peptide, has been reported. CNA is a proven virulence factor in many staphylococcal infections in a plurality of mechanisms. It is commonly known that CNA functions as an adhesin and can mediate colonization onto surfaces. [4] Recently, an alternate mechanism of action was found by inhibiting complement activation which results in evasion of immune responses. [27]

Our bioinformatics analysis results demonstrated the shared N1-N2 domains of CNA across over 50 bacterial genera. The function and structural mechanisms of many bacterial cell wall anchored proteins within this class of adhesins are relatively similar. Our sequence search ultimately was limited by the database of known sequences and would suggest there are many other bacteria with similar sequence matches. Although the matches for non-bacterial taxonomies were lower, a more detailed phylogenetic study could be performed to investigate evolutionary pathways. Also, mutations of some of the residues in the postulated collagen binding trench on CNA abolished or greatly reduced the collagen binding ability of the MSCRAMM. However, these residues are not necessarily conserved in the collagen binding A region of ACE [28] or the recently described RspA and RspB [29], suggesting differences in the detailed binding mechanisms of these molecules. The comparison between similar collagen-binding proteins with associated binding mechanisms and mutational analysis can be investigated further, similar to those seen in literature. [30]

Using a combination of molecular dynamics and normal mode analysis techniques, we examined the feasibility of the collagen hug mechanism and various protein-protein interactions. Using steered molecular dynamics simulations, an *in silico* open-state CNA was formed and allowed to equilibrate. The natural modes exhibited by the N1-N2 domains are consistent with the hinge bending movements required to bind collagen. The linker length and sequence of residues appear as primary factors in inducing the conformational changes necessary to bind to collagen. Further studies into these factors would be especially compelling. The latch sequence from the N2 domain that forms a beta sheet in the groove of the N1 domain of CNA was also investigated through umbrella sampling free energy calculations. We observe that the free energy required to de-latch the closed state is greater than the order of thermal energy fluctuations. Therefore, the latch is required for effective collagen binding and the absence, mutation, or weak-binding of the latch would affect overall collagen adhesion—akin to results from similar proteins such as ACE. [18] Finally, the differing non-bonded interactions, mainly hydrophobic and electrostatic, between the bound CNA and collagen peptides were examined. The binding is initiated by a low-affinity interaction between the collagen triple helix and the complementary shallow trench on the N2 domain and stabilized by hydrophobic and electrostatic interactions. The N1 domain is most probably not involved initially. In the next step, the inter-domain linker interacts with the residues on the collagen chain and contributes to repositioning the N1 domain. The N1 domain is then stabilized by the critical hydrophobic residues. The locking then occurs after being primed by this reorganization to fit the latch within the N1 domain groove.

In the last part of this study, we investigated the structure and binding properties of the collagen-CNA complex while collagen is under tensile-loading. *In vivo*, collagen is subject to a variety of mechanical forces in the ECM. We showed that CNA binds less strongly to stretched than relaxed collagen, implying the mechanoregulation of the bacterial-binding site on collagen.

To confirm with past studies on the biomechanical properties of different types of collagen, single collagen molecules have been shown to be very flexible at physiological conditions. For type I procollagen, its persistence length is less than one-twentieth of molecule length and for type II procollagen it is about one-thirtieth of its molecule length. [31] Experimental studies have shown the relatively facile stretching of a single collagen I monomer by the same length as is its contour length. [31] For larger scales such as tropocollagen, it has been shown that collagen can withstand strains of up to 0.3 before departing from its linear elastic region and losing its helical structure. [32] This would correspond to near 24 nN of tensile loading before approaching fracture for a particular crystallized collagen system- PDB ID 1QSU [33]. In our SMD simulations, the average strain at 800 pN is less than 0.20—which is significantly less than allowable strains from literature. [32] It is important to note though that it is not the direct correlation with force but the force-induced mechanical strain that is the indicator of changes across the structure-function relationship of stretched proteins. Further experimental validation of such a setup would be interesting.

CNA plays important roles in *S*. *aureus* pathogenesis. CNA has been demonstrated to be a virulence factor for *S*. *aureus* infection [4], with virulence being dependent upon the collagen-binding activity of CNA [34]. Furthermore, CNA has been implicated in *S*. *aureus*-mediated osteomyelitis [14], experimental arthritis [34], and corneal infections [5]. Due to its importance in *S*. *aureus* pathogenesis, CNA may also serve as a useful therapeutic target. Antibodies targeted against CNA have been shown to be able to inhibit collagen adhesion of *S*. *aureus* [6,35]. Such an anti-adhesion therapy could promote the detachment of *S*. *aureus* from biofilms, facilitating bacterial clearance. The results of our study could be of potential relevance to future efforts aimed at targeting CNA for the treatment of antibiotic-resistant bacterial infections.

Moreover, the results implicate a selective affinity of bacteria to un-stretched collagen. This study, along with a separate study of a different bacterial adhesin and ECM protein, fibronectin, [2] provides a compelling narrative that mechanical factors modulate bacterial adhesion and subsequent disease progression. Collagen is found in many tissue systems. But particularly at wound sites and areas of inflammation, the collagen fibers are expected to be relaxed. During wound healing, collagen undergoes rapid turnover, with high levels of collagen deposition and cleavage [36]. Newly deposited collagen during wound healing is relatively disorganized and lacks cross-links, which results in collagen relaxation. Furthermore, collagen cleavage by matrix metalloproteinases present during wound healing or inflammation relieves the tension in the collagen network, leading to collagen fiber relaxation. Injured or diseased tissue might thus present collagen in different states, and this could subsequently regulate early adhesion events. These principles are not limited to native tissue but also can be applied to bioprosthetics and engineering solutions that might utilize collagen. It could give further insight into design parameters, notably network tension of collagen, necessary to prevent bacterial infection.

## Materials and methods

### Sequence similarity

We used the FASTA algorithm for the sequence similarity search on database sequences provided by European Bioinformatics Institute at the European Molecular Biology Laboratory [37]. FASTA performs a local heuristic search of a protein or nucleotide database for a query of the same type. We used several databases from the UniProt Knowledgebase (UniProtKB) [23] as the central access point for extensive curated protein information, including function, classification, and cross-references. The specific taxonomic subsets used were for bacteria, eukaryota, viruses, and archaea.

The BLOSUM50 substitution matrix was used for scoring alignments when searching the database. A 25% target identity was used as the average alignment identity the matrix would produce in the absence of homology and can be used to compare different matrix types. Alignment boundaries are more accurate when the alignment identity matches the target identity percentage. We set a gap open penalty, score for the first residue in the gap, of -10 and a gap score for each additional residue of -2. FASTA uses a rapid word-based lookup strategy to speed the initial phase of the similarity search. The KTUP is used to control the sensitivity of the search. Lower values lead to more sensitive, but slower searches. Our KTUP value was 2. We set the expectation upper limit, which corresponds to the maximum number of times the match is expected to occur by chance, to 10.

### Initial structure and configuration

PDB ID 2F6A was used for the CNA_31-344_ protein and the collagen peptide. Nariyana et al crystallized both an apo-CNA_31-344_ and CNA_31-344_ structure in complex with a collagen peptide [16]. The apo-form of CNA is hypothesized to exist in some balance between open and closed conformation with only the open conformation able to bind to collagen. The crystal structure of apo-CNA_31-344_ in PDB ID 2F68 was in the closed conformation. The N1 and N2 domains of CNA are connected by a long linker region. Unfortunately in PDB ID 2F68, the linker region was missing residues and was not suitable for our purposes. We therefore instead used PDB ID 2F6A for both the CNA N1-N2 complex and collagen peptide. The crystal structure of the CNA-collagen complex resembles a dumbbell, with two CNA molecules bound at each end of the collagen peptide. Using visual molecular dynamics (VMD), we selected one of these CNA molecules along with an adequate length of the collagen peptide (~45 Å) for our isolated studies. CNA can bind to many sites along collagen. Sixteen synthetic collagen peptides, based on the alpha-1 chain of bovine or chicken type I collagen, were already screened with varying amounts of GPO or GPP triplets (with O signifying an important non-standard amino acid hydroxyproline used in stabilization of the collagen structure). The crystallized synthetic collagen forms three (GPO)_4_GPRGRT(GPO)_4_ strands in a right handed triple helix with an approximate length of 90 Å. Lastly, all molecular dynamics simulations were using NAMD [38] with the CHARMM27 force field [39] extended for the hydroxyproline (HYP) residue [40,41].

### Steered molecular dynamics for collagen under tension

In general, the all-atom simulations were performed using NAMD, a molecular dynamics software with a CHARMM27 force field and TIP3P explicit water model [38]. All charges on the protein were neutralized via the addition of K+ or Cl- ions, and KCl was added to the system to reach a concentration of 150 mM. A timestep of 2 femtoseconds was utilized for all simulations, and the LINCS algorithm was used to constrain the bonds between hydrogens and heavy atoms. Electrostatic interactions were calculated using the particle mesh Ewald method. All post-simulation visualization and analysis were performed using Visual Molecular Dynamics (VMD) software [42].

The bound CNA-collagen complex was initially minimized for 50000 steps, using the conjugate gradient and line search algorithm implemented in NAMD [38]. Following minimization, the configuration was simulated for 3 ns, until equilibrium was reached. The root mean square deviation (RMSD) of the atomic positions was used to check equilibrium state.

A series of constant force pulling simulations were then performed on the equilibrated CNA-collagen setup. In these steered molecular dynamics (SMD) simulations, the alpha carbons at one end of the collagen peptides is fixed while the other end is pulled along a parallel vector in order to elongate the synthetic collagen. A range of forces, 80, 400, 600, 800, 4000, and 8000 pN were applied. Each one of these simulations ran for at least 10 ns, with two simulations ran for 40 ns to examine if any conformations occur past the 10 ns time frame. In order to establish statistical significance while keeping the computational constraints in mind, we repeated every simulation in this study three times. The structures from each of the trials are examined and compared in VMD—along with quantification of the RMSD and energy changes.

### Interaction energy calculations

For the latch disassociation, the interaction energies—electrostatic, van der Waals, and combined energies—of the latch and N1 domain of CNA were calculated over the course of the SMD simulation. For the collagen under tension SMD simulations, the interaction energy between CNA N1-N2 and collagen were calculated over the course of each respective simulation. The energy values are the sum of the non-bonded interactions—namely electrostatic and van der Waals. For the box plot statistics, the interaction energy values were used only after stabilization of the interaction energy curve versus time.

### Steered molecular dynamics to induce *in silico* open state CNA

Constant force Steered Molecular Dynamics (SMD) was used to computationally induce the open state of apo-CNA (PDB ID: 2F6A [17]). apo-CNA was minimized for 50,000 steps and equilibrated until stabilization of its root mean square deviation (RMSD). The system was solvated, neutralized, and ionized to a concentration of 150 mM using KCl. SMD was conducted in two stages: 1) Dissociation of the latch from domain N2 of CNA, and 2) separation of domains N1 and N2 of CNA. For the first stage, the alpha carbons of residue 318 near the latch and residues 122–133 of N1 were fixed, and a constant force of 800 pN was applied to the alpha carbon of residue 330 to separate the latch and N2 in apo-CNA. For the second stage, the alpha carbon of residues 165 (which flanks the N1-N2 linker) and 88–89 (which are in spatial proximity to N2) in N1 were fixed, and 100 pN was applied to the each alpha carbon of residues 177–179 in N2 to separate N1 and N2. The system was equilibrated following SMD. Simulations were performed using the Molecular Dynamics software package NAMD [38], and all post-simulation visualization and post-processing was performed using Visual Molecular Dynamics software [42].

### Normal mode analysis

Normal mode analysis (NMA) was performed to examine the natural frequencies exhibited by the protein. WEBnm@, an online normal mode analysis tool, calculates the eigenvectors of the matrix of second derivatives of energy with respect to displacement of the alpha-C atoms of each residue [43]. Because NMA represents movements resulting from overall structure, the use of alpha-C force fields is sufficient for NMA calculation. The lowest frequency normal modes were taken to observe the conformational movements and vibrational frequencies. Vector fields of the vibrational movements were produced by WEBnm@ and examined in VMD along with a new ribbon representation to illustrate the modal movements. We performed NMA on both the closed-state CNA and the induced open-state CNA. The first eight mode were static and therefore not used. The next 9–18 modes were examined to deduce the natural conformational movements exhibited by CNA. A similar simulation was run on the AD-ENM (Analysis of Dynamics of Elastic Network Model) server which performs analysis of macromolecular dynamics based on a highly simplified physical model called the Elastic Network Model (ENM). [44] It served as a confirmation of the conformational modes observed via WEBnm@.

### Umbrella sampling

Umbrella Sampling was performed to obtain the potential of mean force (PMF) [45]. The reaction coordinate used for sampling was the distance between the center of mass of the latch (residues 320–330) and domain N2 (residues 31–140). 10 ns sampling was performed for each umbrella. A total of 25 umbrellas were included in the PMF calculation, and umbrellas were variably spaced relative to each other to optimize overlap, but were typically spaced approximately 0.15 nm apart. The PMF was obtained using Grossfield’s WHAM [46] in GROMACS software [47]. For the initial steered molecular dynamics, the N2 latch was pulled away from domain N1 (domain N2, with the exception of the N2 latch, was not included in simulations), where a pulling velocity of 0.01 nm/ps was applied.

## Supporting information

S1 Fig Demonstrated contrast of non-linear behavior for high, non-physiologically relevant forces at 4000 pN-8000 pN vs. linear behavior for relevant forces at 800pN and below. (A) The average strain vs. load curve demonstrating the force dependence on length elongation of collagen. (B) Calculated average bond energies for the individual collagen peptides. Plot is used to separate the 80–800 pN physiologically relevant forces from the improbable 4000 pN and 8000 pN applied forces as they strain bonds.
(TIF)
