# Supplementary figures and images for: Molecular mechanics of Staphylococcus aureus adhesin, CNA, and the inhibition of bacterial adhesion by stretching collagen
Source: PLoS One. 2017 Jun 30;12(6):e0179601. doi: 10.1371/journal.pone.0179601 (PMC5493303; doi:10.1371/journal.pone.0179601)

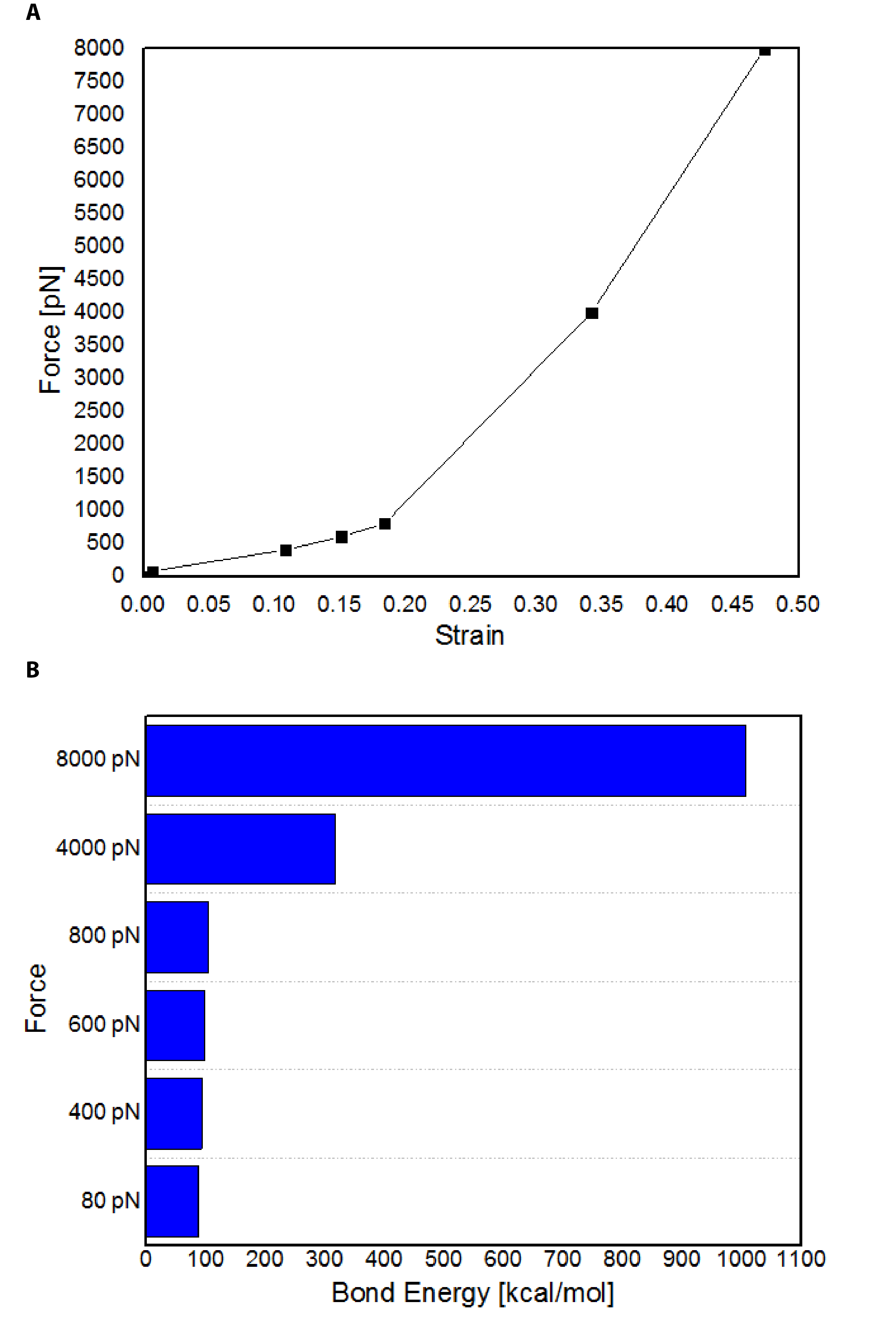

Supplement: S1 Fig — (A) The average strain vs. load curve demonstrating the force dependence on length elongation of collagen. (B) Calculated average bond energies for the individual collagen peptides. Plot is used to separate the 80–800 pN physiologically relevant forces from the improbable 4000 pN and 8000 pN applied forces as they strain bonds. (TIF) [file pone.0179601.s001.tif]
